# Trigonelline Improves Metabolism and Cardiac Function of HFpEF Mice Via Gut Microbiome Alterations‐Mediated AMPK Activation

**DOI:** 10.1002/advs.202513956

**Published:** 2025-11-05

**Authors:** Zhe Cheng, Xiaowen Wang, Jiang Yu, Xiaorong Li, Qing Tang, Can Qu, Ding Yang, Yuanjing Li, Yong Xia, Yongzheng Guo

**Affiliations:** ^1^ Department of Cardiovascular Medicine, Cardiovascular Research CenterCenter The First Affiliated Hospital of Chongqing Medical University Chongqing 400016 China; ^2^ Division of Cardiology Chongqing University Three Gorges Hospital Chongqing 400016 China; ^3^ Department of Cardiothoracic Surgery The First Affiliated Hospital of Chongqing Medical University Chongqing 400016 China; ^4^ School of Basic Medicine Science Chongqing Medical University Chongqing 400016 China; ^5^ Department of Pharmacy The First Affiliated Hospital of Chongqing Medical University Chongqing 400016 China

**Keywords:** AMPK, gut microbiota, HFpEF, non‐targeted metabolomics, trigonelline

## Abstract

Heart failure with preserved ejection fraction (HFpEF) is a prevalent end‐manifestation of cardiovascular diseases currently lacking effective treatment. Using a high‐fat diet and L‐NAME‐induced mouse model, untargeted metabolomic profiling is performed and trigonelline is identified as a markedly reduced metabolite in HFpEF hearts. Oral trigonelline supplementation alleviates metabolic syndromes, including obesity, insulin resistance, and hepatic injury, leading to improved cardiac function in HFpEF mice. AMPK inhibition blunts the protective effects of trigonelline despite trigonelline per se not activating AMPK directly. Gut microbiota is required in AMPK activation and consequent beneficial effects on HFpEF mice by trigonelline. Further investigations demonstrate that trigonelline significantly restores HFpEF mouse gut microbiome dysbiosis by decreasing Firmicutes and increasing Bacteroidetes. In conclusion, the studies demonstrate that trigonelline supplementation mitigates HFpEF‐associated metabolic disorders and improves cardiac function via gut microbiome alterations‐mediated AMPK activation. These findings suggest that trigonelline has therapeutic potential for HFpEF.

## Graphical abstract

Metabolomics analyses reveal that trigonelline is markedly reduced in HFpEF hearts. Trigonelline supplementation alleviates metabolic disorders and improves cardiac function in HFpEF mice via gut microbiota‐mediated AMPK activation.

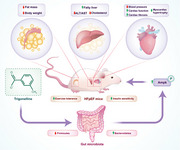

## 1 Introduction

Heart failure with preserved ejection fraction (HFpEF) accounts for nearly half of all heart failure cases and carries high morbidity and mortality.^[^ ^1^ ^]^ Due to the lack of evidence‐based drug interventions, the 5‐year survival rate after the first hospitalization of HFpEF is only ≈35–40%.^[^ ^2^, ^3^ ^]^ The molecular and biological mechanisms underlying HFpEF are highly complicated.^[^ ^4^, ^5^ ^]^ Previous studies, including ours, show that metabolic inflexibility, inflammation, nitrosative/oxidative stress, mitochondrial dysfunction, and an imbalance in nitric oxide (NO) levels all contribute to HFpEF pathogenesis.^[^ ^6^, ^7^, ^8^, ^9^, ^10^, ^11^ ^]^

AMP‐activated protein kinase (AMPK) serves as a master regulator of cellular energy homeostasis, orchestrating glucose and lipid metabolism while maintaining mitochondrial function.^[^ ^12^ ^]^ In HFpEF, AMPK activity is often suppressed, which exacerbates metabolic stress and cardiac dysfunction.^[^ ^13^ ^]^ Therefore, restoring AMPK activity has emerged as a promising therapeutic approach for HFpEF.

Increasing evidence shows that the disruption of gut microbiota equilibrium is vital in the development of metabolic disorders and cardiovascular diseases, including heart failure.^[^ ^14^, ^15^, ^16^, ^17^, ^18^ ^]^ Among various microbes, Firmicutes and Bacteroidetes account for most of the gut microbiota,^[^ ^19^, ^20^ ^]^ and the Firmicutes/Bacteroidetes (F/B) ratio has been employed to evaluate gut microbiota dysbiosis.^[^ ^21^ ^]^ Indeed, increased F/B ratios are associated with metabolic disorders, obesity, and insulin resistance.^[^ ^22^, ^23^ ^]^ Yet the potential roles of gut microbiota in the development of HFpEF are still largely unclear.

Trigonelline is a naturally occurring alkaloid derived from dietary sources, and is also modulated by gut microbial metabolism.^[^ ^24^, ^25^ ^]^ Previous studies have suggested that trigonelline exerts multiple biological effects, including anti‐inflammatory, antioxidant, and metabolic regulatory properties, with potential cardiovascular benefits.^[^ ^26^, ^27^ ^]^ Since gut microbiota can influence the whole‐body metabolic activities, and trigonelline involved into metabolic regulatory, it would be intriguing to determine if trigonelline can modulate the metabolism and function of HFpEF hearts via intervening gut microbiota equilibrium.

In this study, we induced HFpEF in mice by exposing them to high‐fat diet (HFD) and N‐nitro‐L‐arginine methyl ester (L‐NAME) treatments.^[^ ^11^ ^]^ Our non‐targeted metabolomics analyses on heart tissues uncovered trigonelline was the most decreased metabolite in HFpEF hearts. Oral trigonelline supplementation alleviated metabolic syndromes, leading to improved cardiac function in HFpEF mice. Further mechanistic studies revealed that trigonelline improved the metabolism and cardiac function of HFpEF mice via gut microbiome alterations‐mediated AMPK activation.

## 2 Results

### 2.1 Untargeted Metabolomics Revealed a Distinctive Metabolic Profile in HFpEF Hearts

**Figure 1:**
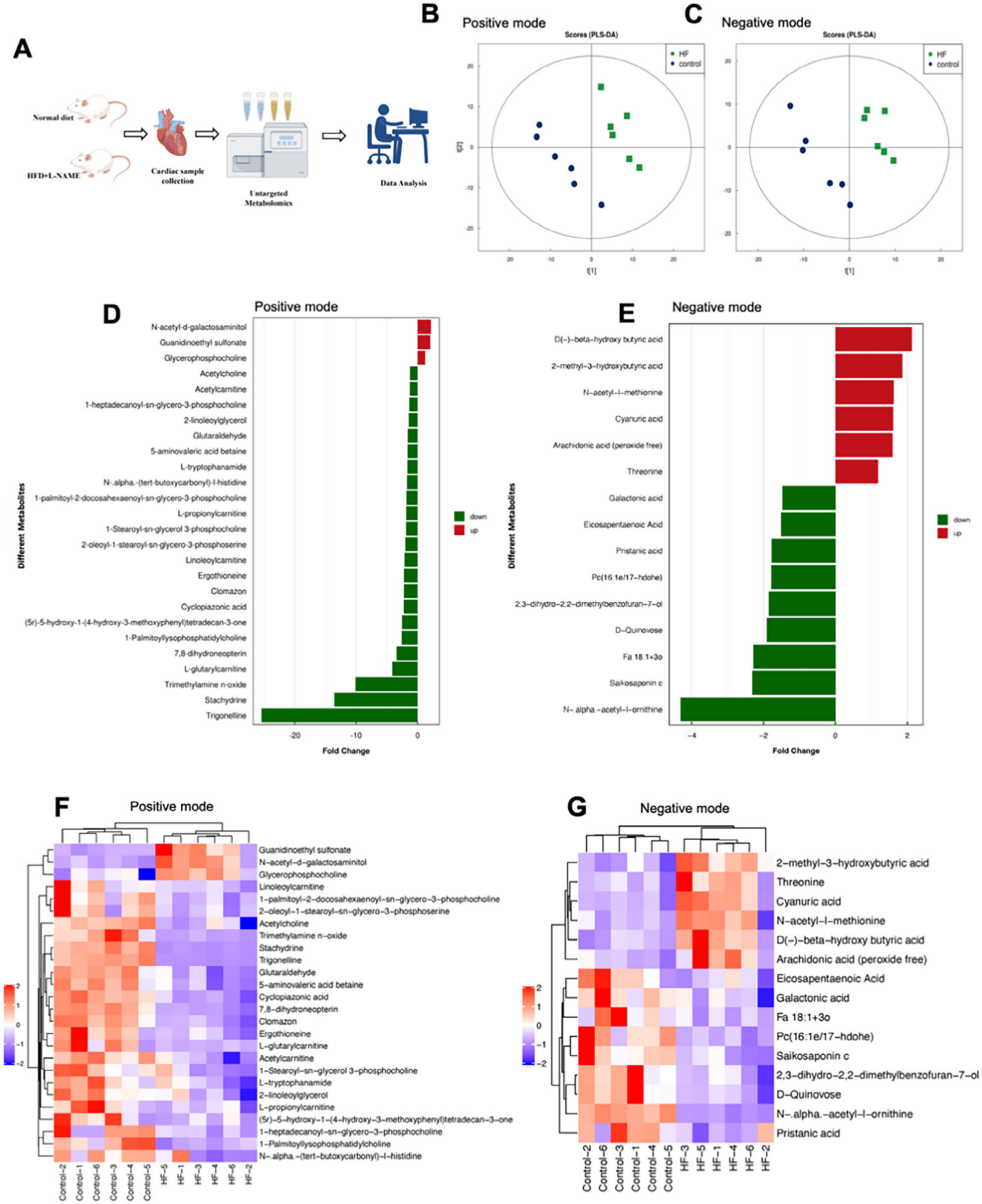
Metabolomics profiling identifies trigonelline as a potential key metabolite in HFpEF mice. A) Schematic showing the metabolomics‐based experimental design using heart samples collected from control and HFD + L‐NAME‐induced HFpEF mice. B,C) The partial least squares‐discriminant analysis (PLS‐DA) score plot shows group distribution based on metabolite profiles of heart tissues of mice in each group. D,E) The histogram of fold change represented a significant difference in the abundance of metabolites between the control and HFpEF groups in each mode. F,G) Heatmap generated by the one‐to‐one fold changes of metabolites with VIP value more than one by PLS‐DA analysis in the untargeted metabolomics dataset of heart tissue samples of HFpEF mice in each mode (*n* = 6).

We performed non‐targeted metabolomic analyses on six pairs of heart tissue samples from control and HFpEF mice to investigate metabolomic profile changes in HFpEF hearts (**Figure** **1**A). The resulting metabolic profiles were clearly segregated between the two groups based on PLS‐DA analysis in ESI‐positive (Figure 1B) and ESI‐negative modes (Figure 1C). PLS‐DA was validated by a permutation test (Figure S1A,B, Supporting Information). The significantly upregulated and downregulated metabolites (OPLS DA VIP>1 and *p*‐value < 0.05) were visualized by a volcano plot (Figure S1C,D, Supporting Information). Detailed information on differential metabolites is presented in Datasets S1 and S2 (Supporting Information), and the metabolic proximities analysis results are shown in, Figure S2 (Supporting Information).

Among all differential metabolites identified from ESI‐positive and ESI‐negative modes, trigonelline exhibited the greatest fold change, nearly a 25‐fold reduction in HFpEF compared with controls (Figure 1D,E). The hierarchical cluster analysis is visualized by a heatmap in Figure 1F,G. These results revealed the distinguished metabolic profile in HFpEF hearts and showed that the trigonelline reduction was consistent among the HFpEF mice.

### 2.2 Trigonelline Supplementation Reversed Cardiac Dysfunction in HFpEF Mice

Beyond its substantial alteration, trigonelline has been reported to alleviate metabolic disorders in diabetes, cardiovascular disorders, and aging,^[^ ^26^, ^27^ ^]^ which are highly relevant to the pathophysiological process of HFpEF. Thus, we next investigated whether trigonelline supplementation showed beneficial effects in HFpEF mice.

**Figure 2:**
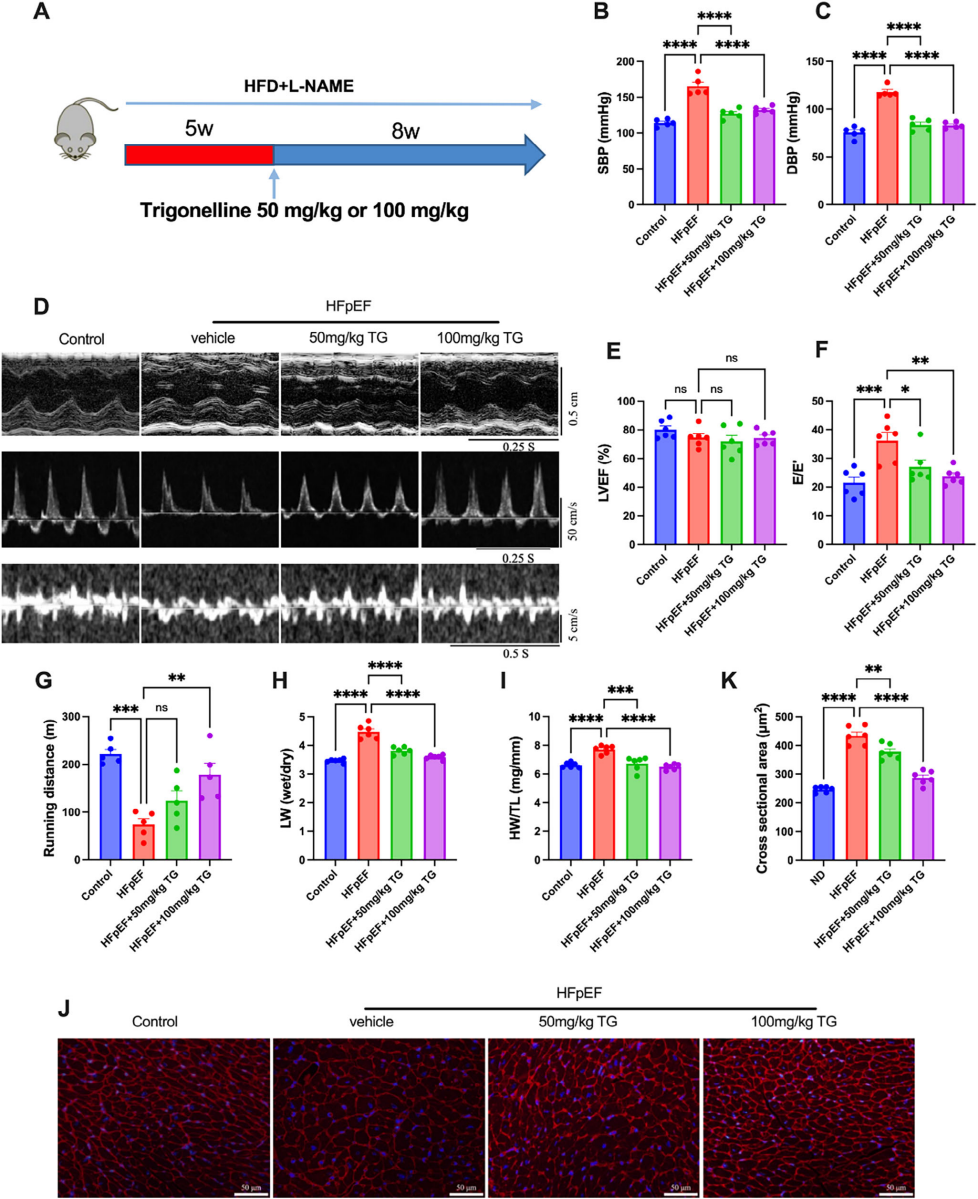
Trigonelline supplementation reversed cardiac dysfunction in HFpEF mice. A) Scheme for the experimental strategy in HFpEF mice treated with trigonelline (50 or 100 mg kg^−1^ day^−1^). B,C) SBP and DBP of different experimental groups, noninvasively measured by the tail‐cuff method (*n* = 5 mice per group). D) Representative echocardiography‐derived M‐mode tracings (top), pulsed‐wave Doppler (middle), and tissue Doppler (bottom) tracings. E) Percent left ventricular ejection fraction (LVEF%). F) Ratio between mitral E wave and E’ wave (E/’E’). G) Running distance during the exercise exhaustion test of mice. H) The ratio between wet and dry lung weight (LW). I) Ratio between heart weight and tibia length (HW/TL) (for LVEF%, E/E’ ratio, running distance, LW wet/LW dry ratio, and HW/TL ratio, *n* = 6 mice per group). J) Representative images of WGA in transversal sections of the left ventricle of mice of different experimental groups. Scale bar: 50 µm for WGA. K) WGA quantification of cardiomyocyte cross‐sectional area (*n* = 6 mice per group). Data are presented as mean ± SEM and analyzed using one‐way ANOVA followed by Tukey's multiple comparisons test. ns, no significant; ^*^ *p* < 0.05, ^**^ *p* < 0.01, ^***^ *p*<0.001 and ^****^ *p* < 0.0001.

As shown in **Figure** **2**A–C, trigonelline supplementation (50 or 100 mg kg^−1^ for eight weeks) significantly reduced systolic and diastolic pressures in HFpEF mice. Trigonelline supplementation alleviated the diastolic dysfunction—a hallmark feature of HFpEF, and showed no significant effects on systolic function and cardiac remodeling (Figure 2D–F; Figure S3, Supporting Information). With improved cardiac function, the exercise intolerance (Figure 2G) and lung edema (Figure 2H) of HFpEF were also significantly improved by trigonelline. Moreover, trigonelline also reduced hypertrophy, as evidenced by a reduced ratio of heart weight to tibial length (Figure 2I) and cross‐sectional area of cardiomyocytes by wheat germ agglutinin (WGA) staining (Figure 2J,K). These data demonstrated that trigonelline supplementation improved cardiac function of HFpEF mice.

### 2.3 Trigonelline Supplementation Alleviated Metabolic Disorders in HFpEF Mice

**Figure 3:**
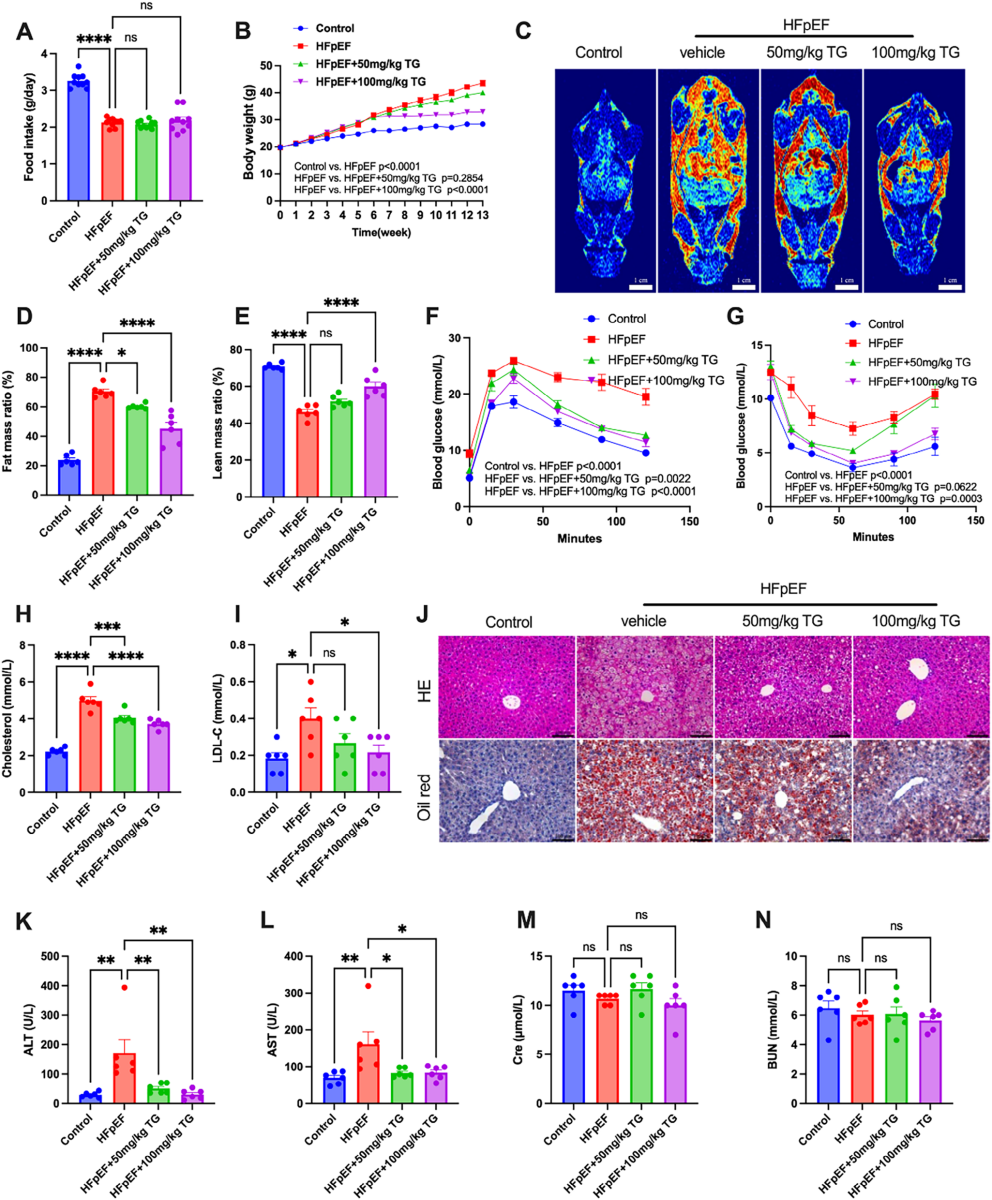
Trigonelline supplementation alleviated metabolic disorders and hepatic injury in HFpEF mice. A) Food intake of mice of different experimental groups per day (*n* = 10). B) Body weight was monitored weekly in each experimental group (*n* = 10). C) Representative images of the MRI of mice of different experimental groups. Red indicates higher fat content, green represents moderate fat content, and blue corresponds to low fat content. Scale bar: 1 cm. D,E). Fat mass and lean mass ratios of mice in the indicated groups. F) Glucose tolerance tests in the indicated groups (*n* = 5). G) Insulin sensitivity tests in the indicated groups (*n* = 5). H) Total cholesterol of serum in each group. I) Low‐density lipoprotein cholesterol (LDL‐C) in each group. J) Representative images of hematoxylin and eosin staining and oil red staining of liver from mice in each treated group, Scale bar = 100 µm. K) Serum ALT (glutamic oxaloacetic acid transferase) in each group. L) Serum AST (glutamic‐pyruvic transaminase) in each group. M) Serum Cre (creatinine) in each group. N) Serum BUN (urea nitrogen) in each group (for total cholesterol, LDL‐C, Serum ALT, AST, Cre, and BUN, *n* = 6 mice per group). Data are presented as mean ± SEM and analyzed using one‐way ANOVA followed by Tukey's multiple comparisons test. ns, no significant; ^*^ *p* < 0.05, ^**^ *p* < 0.01, ^***^ *p* < 0.001, and ^****^ *p* < 0.0001.

Metabolic disorders often precede the onset of HFpEF and have been shown to be an important mechanism driving the failure of cardiac function.^[^ ^5^ ^]^ We thus evaluated the effects of trigonelline supplementation on metabolic phenotypes in HFpEF mice. Although trigonelline did not affect the food intake of HFpEF mice (**Figure** **3**A), 100 mg kg^−1^ trigonelline supplementation for eight weeks significantly reduced body weight gain (Figure 3B) and fat accumulation (Figure 3C–E) in HFpEF mice. Trigonelline also ameliorated insulin resistance, as evidenced by glucose tolerance (Figure 3F) and insulin sensitivity tests (Figure 3G). In addition to insulin resistance, trigonelline supplementation mitigated dyslipidemia in HFpEF mice (Figure 3H,I). Trigonelline reduced lipid droplets in liver cells (Figure 3J) and decreased ALT (Figure 3K) and AST (Figure 3L) levels in the serum of HFpEF mice, thus alleviating hepatic steatosis. The creatinine (Figure 3M) and blood urea nitrogen (Figure 3N) levels were slightly affected by trigonelline. Together, these data demonstrated that trigonelline effectively ameliorated metabolic disorders in HFpEF mice, which might account for improved cardiac function.

### 2.4 AMPK Activation was Required for the Cardioprotective Effects of Trigonelline in HFpEF Mice

**Figure 4:**
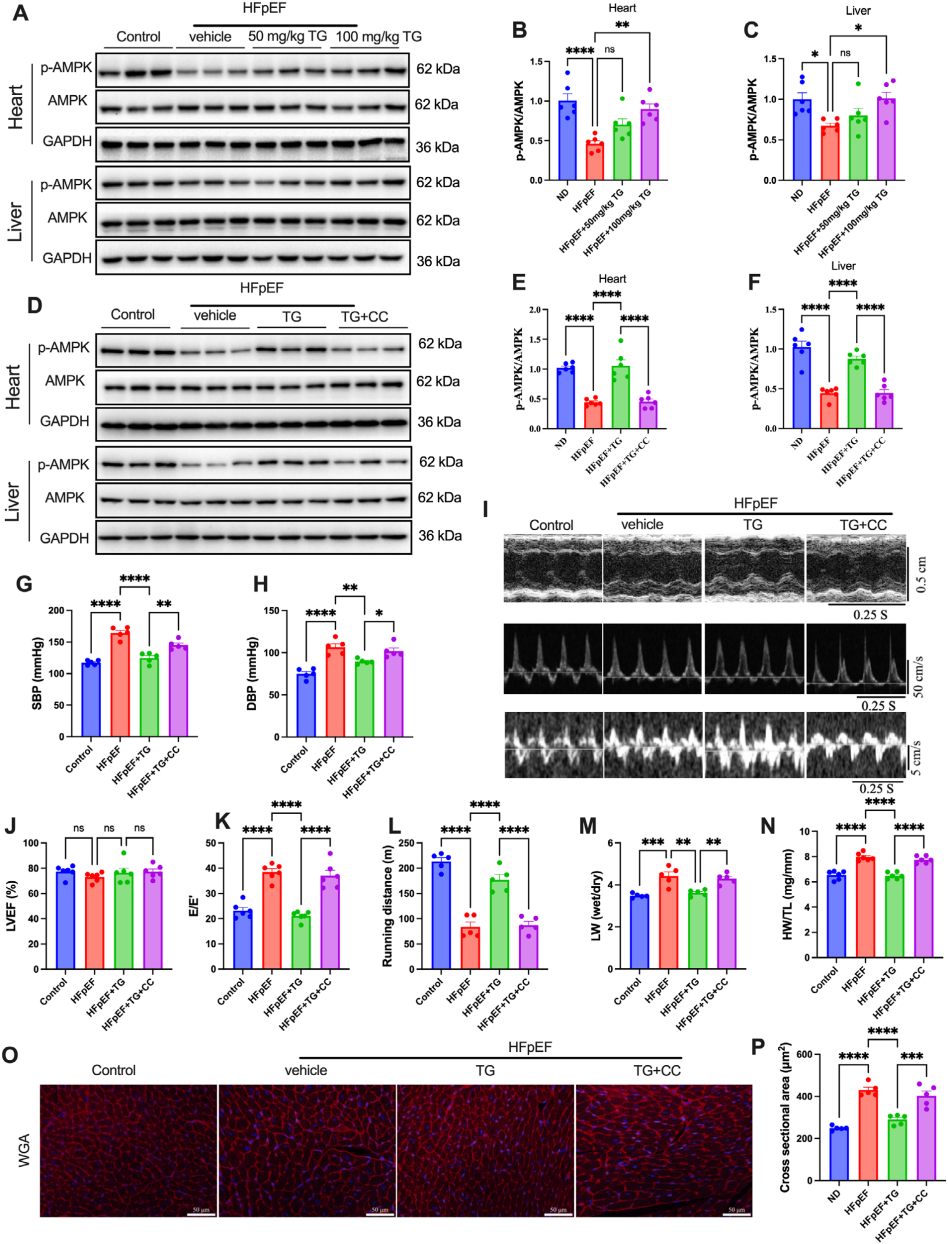
AMPK activation was required for the cardioprotective effects of trigonelline in HFpEF mice. A) Representative immunoblot images of total and phosphorylated AMPK in the heart and liver tissues from HFpEF mice receiving trigonelline. B,C) Quantification of cardiac p‐AMPK/AMPK (*n* = 6 mice per group). D) Representative immunoblot images of total and phosphorylated AMPK in the heart and liver tissues from HFpEF mice receiving trigonelline with or without AMPK inhibitor. E,F) Quantification of hepatic p‐AMPK/AMPK ratio (*n* = 6 mice per group). G,H) SBP and DBP of different experimental groups (*n* = 5 mice per group). I) Representative echocardiography‐derived M‐mode tracings (top), pulsed‐wave Doppler (middle), and tissue Doppler (bottom) tracings of mice in the indicated group. J) Percent left ventricular ejection fraction (LVEF%). K) The ratio between mitral E wave and E’ wave (E/E’). L) Running distance during the exercise exhaustion test of mice. M) The ratio between wet and dry lung weight (LW). N) Ratio between heart weight and tibia length (HW/TL) (for LVEF%, E/E’ ratio, running distance, LW wet/LW dry ratio, and HW/TL ratio, *n* = 6 mice per group). O) Representative images of WGA in transversal sections of the left ventricle of mice of different experimental groups. Scale bar: 50 µm for WGA. P) WGA quantification of cardiomyocyte cross‐sectional area (*n* = 5 mice per group). Data are presented as mean ± SEM and analyzed using one‐way ANOVA followed by Tukey's multiple comparisons test. ns, no significant; ^*^ *p* < 0.05, ^**^ *p* < 0.01, ^***^ *p* < 0.001 and ^****^ *p* < 0.0001.

AMPK is a predominant sensor and modulator in intracellular energy and metabolism regulation.^[^ ^28^, ^29^, ^30^ ^]^ AMPK dysregulation has been implicated in various metabolic cardiac injuries. We therefore investigated whether trigonelline's cardioprotective actions on HFpEF mice were associated with AMPK activation. AMPK is primarily activated by the phosphorylation of its Thr172 residue.^[^ ^29^, ^30^ ^]^ As shown in **Figure** **4**A–C, decreases in phospho‐AMPK were seen in both hearts and livers of HFpEF mice, indicating that dysregulated metabolic signaling occurred. Trigonelline supplementation dose‐dependently increased cardiac and hepatic AMPK phosphorylation. Interestingly, trigonelline did not induce the AMPK activation in kidneys and skeletal muscles (Figure S4, Supporting Information). These findings indicated that HFpEF mice had downregulated AMPK activity in hearts and livers. Trigonelline could restore AMPK activation in both hearts and livers in HFpEF mice.

To examine whether trigonelline protects against HFpEF via activating AMPK, we treated mice with an AMPK inhibitor, compound C,^[^ ^28^ ^]^ to inhibit AMPK activity, as presented in Figure S5A (Supporting Information). As expected, compound C reversed the effects of trigonelline on the heart and liver AMPK in HFpEF mice (Figure 4D–F). And considering the previous evidence that GSK‐3β and AMPK exert opposing roles in cellular energy homeostasis, we also examined the phosphorylation status of GSK‐3β in HFpEF mouse hearts. HFpEF mice exhibited significantly decreased phosphorylation of GSK‐3β (Figure S5B–E, Supporting Information). Oral trigonelline supplementation markedly enhanced GSK‐3β phosphorylation in HFpEF hearts. Notably, this effect of trigonelline on GSK‐3β phosphorylation was reversed by the AMPK inhibitor compound C (Figure S5B–E, Supporting Information), suggesting that trigonelline regulates GSK‐3β phosphorylation in an AMPK‐dependent manner.

Moreover, the protective effects of trigonelline on SBP, DBP, and cardiac function of HFpEF mice were largely reversed by AMPK inhibition (Figure 4G–K; Figure S6, Supporting Information). Moreover, the beneficial effects of trigonelline on exercise intolerance, lung edema, and cardiac hypertrophy were all abolished by AMPK inhibition (Figure 4L–P). Together, these results demonstrated that the cardioprotective effects of trigonelline in HFpEF mice were AMPK‐dependent.

### 2.5 AMPK Inhibition Abolished the Beneficial Effects of Trigonelline on Metabolic Disorders in HFpEF Mice

**Figure 5:**
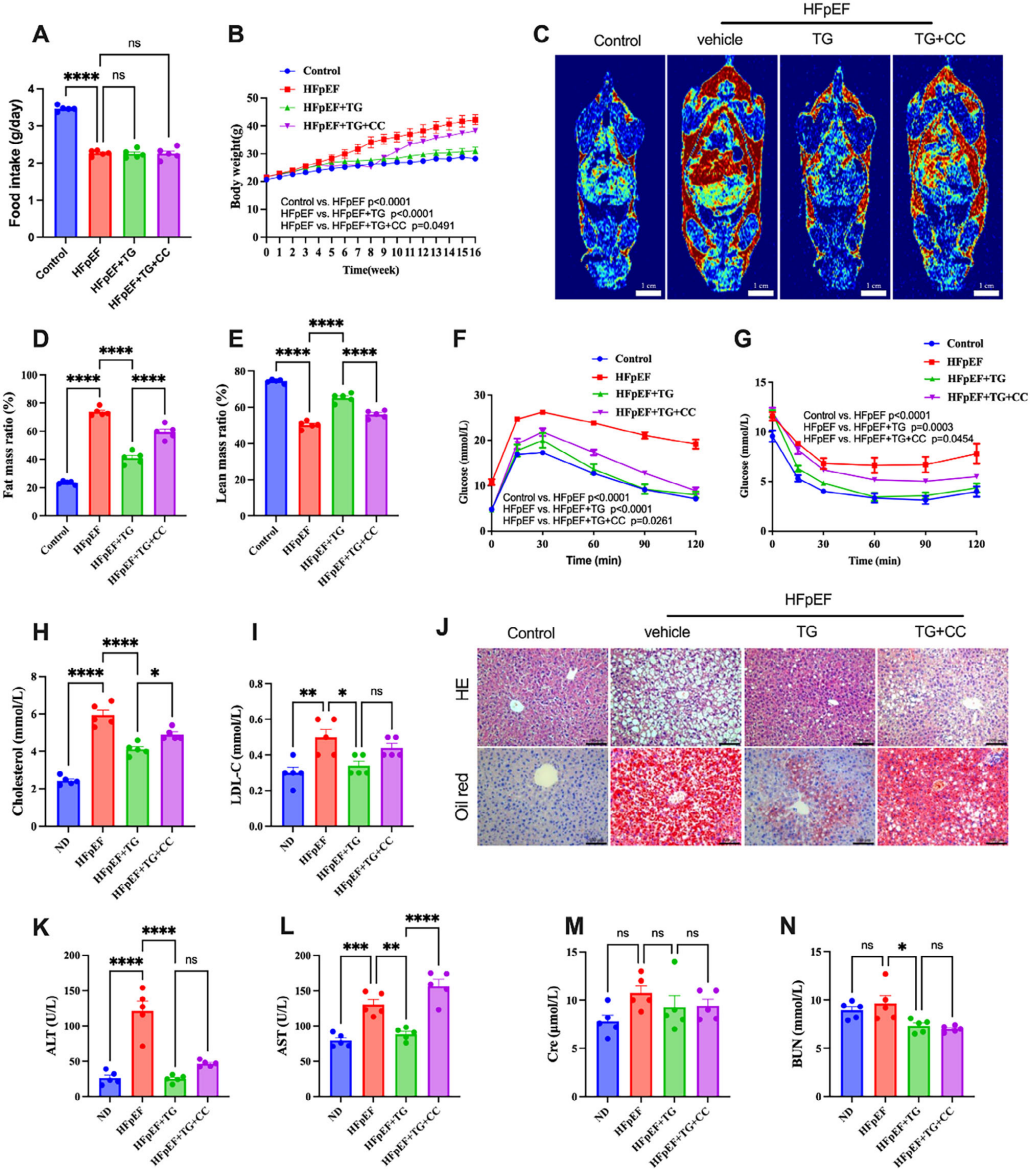
AMPK inhibition abolished the beneficial effects of trigonelline on metabolic disorders in HFpEF mice. A) Food intake of mice per day of different experimental groups per day (*n* = 5). B) Body weight was monitored weekly in each experimental group. C) Representative images of MRI of mice from different experimental groups. Red indicates higher fat content, green represents moderate fat content, and blue corresponds to low fat content. Scale bar: 1 cm. Fat mass D) and lean mass E) ratios of mice in the indicated groups (*n* = 5). F) Glucose tolerance tests in the indicated groups (*n* = 5). G) Insulin sensitivity tests in the indicated groups (*n* = 5). H) Total cholesterol of serum in each group. I) Low‐density lipoprotein cholesterol (LDL‐C) of the serum in each group. J) Representative images of hematoxylin and eosin staining and oil red staining of liver from mice in each treated group, Scale bar = 100 µm. K) Serum ALT in each group. L) Serum AST in each group. M) Serum Cre in each group. N) Serum BUN in each group (for total cholesterol, LDL‐C, Serum ALT, AST, Cre, and BUN, *n* = 5 mice per group). Data are presented as mean ± SEM and analyzed using one‐way ANOVA followed by Tukey's multiple comparisons test. ns, no significant; ^*^ *p* < 0.05, ^**^ *p* < 0.01, ^***^ *p* < 0.001, and ^****^ *p* < 0.0001.

To determine if the effects of trigonelline on metabolic disorders were also due to AMPK activation, similar studies were carried out by measuring the metabolic changes of HFpEF mice with or without compound C treatment. As shown in **Figure** **5**A,B, AMPK inhibition blunted the attenuation of body weight gain by trigonelline in HFpEF mice without affecting their food intake. Moreover, AMPK inhibition significantly abrogated the protective effects of trigonelline on fat accumulation, insulin resistance, and hyperlipidemia (Figure 5C–I). The protective effects of trigonelline on hepatic steatosis were also reversed by AMPK inhibition (Figure 5J–L), without affecting the creatinine (Figure 5M) and blood urea nitrogen (Figure 5N) levels in HFpEF mice. These results indicated that the protective effects of trigonelline on metabolic disorders of HFpEF mice were also AMPK‐dependent.

### 2.6 Trigonelline Supplementation Altered Gut Microbiota of HFpEF Mice, and this was Required for its Action on AMPK

We then sought to decipher how trigonelline induced AMPK activation in HFpEF mice. We first exposed cultured cardiomyocytes (HL‐1) and hepatic cells (HepG2) to increasing concentrations of trigonelline to determine if trigonelline could directly activate AMPK. Interestingly, trigonelline failed to induce AMPK activation in either cultured myocytes or hepatic cells (Figure S7, Supporting Information). These data hinted that trigonelline induced AMPK activation in HFpEF mice through an indirect mechanism.

**Figure 6:**
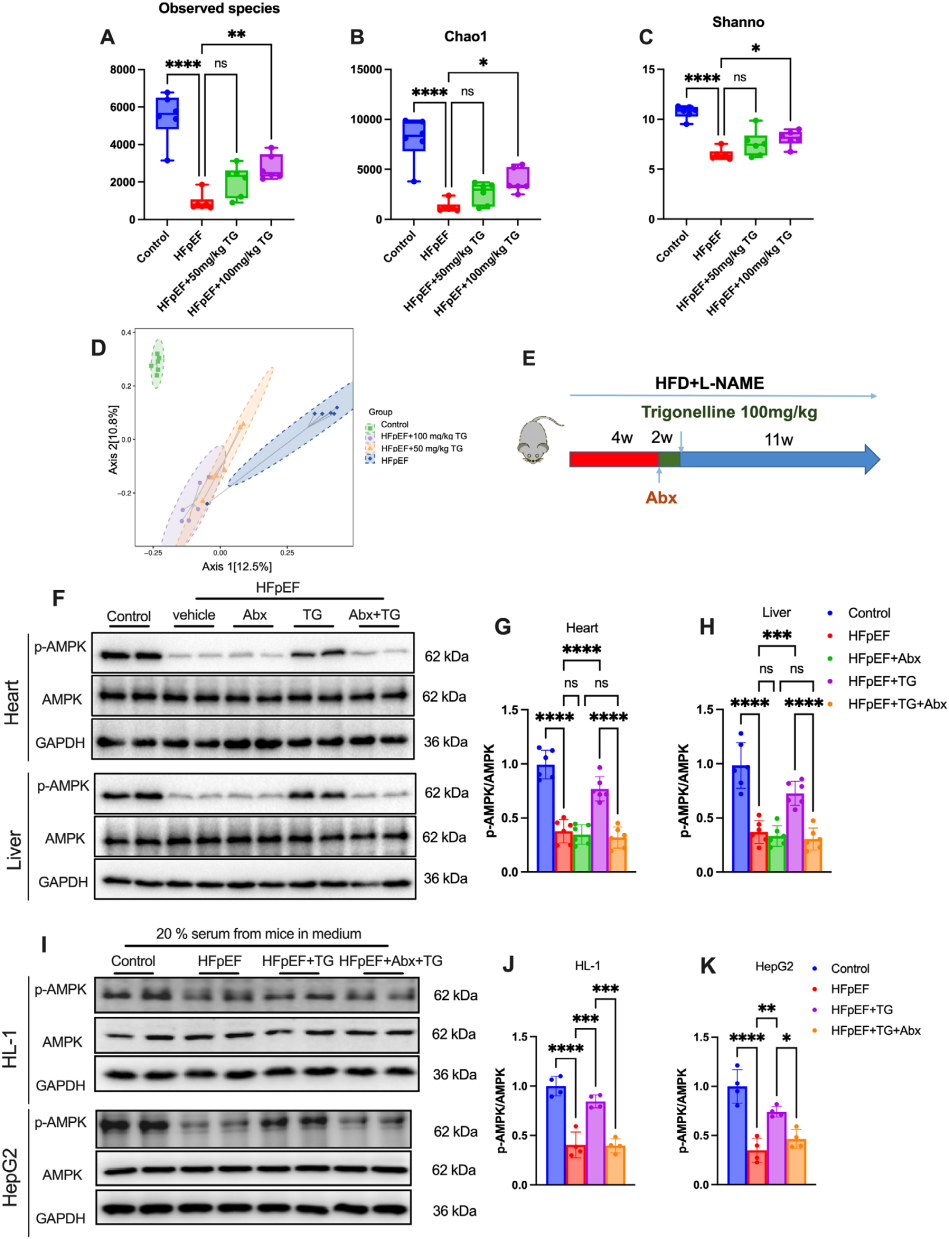
Trigonelline activates AMPK in a gut microbiota‐dependent manner. A–C) Diversity of the gut microbiota in each group, as indicated by the observed species, Shannon, and Chao1 indices. D) PCA score plot analysis based on the relative abundance of OTUs. E) Scheme for the experimental strategy in HFpEF mice treated with trigonelline and antibiotics. F) Representative immunoblot images of total and phosphorylated AMPK in the heart and liver tissues of mice in each group. Quantification of cardiac pAMPK/AMPK ratio in the heart G) and liver H) tissues of mice in each group. (*n* = 6 mice per group). I) Representative immunoblot images of total and phosphorylated AMPK in HL‐1 and HepG2 cell lines in each group. Quantification of cardiac pAMPK/AMPK ratio in the HL‐1 J) and HepG2 cell lines K) in each group. (*n* = 4 mice per group). Data are presented as mean ± SEM and analyzed using one‐way ANOVA followed by Tukey's multiple comparisons test. ns, no significant; ^*^ *p* < 0.05, ^**^ *p* < 0.01, ^***^ *p* < 0.001, and ^****^ *p* < 0.0001. Abx, antibiotic cocktail.

Gut microbiota has been increasingly recognized to play crucial roles in metabolic regulation. Changes in gut microbiota has been reported to induce AMPK activation.^[^ ^31^, ^32^ ^]^ We thus hypothesized that trigonelline might induce AMPK activation via altering gut microbiota. To test this hypothesis, we first investigated the effects of trigonelline on the community structure of gut microbiota in HFpEF mice by using 16S rRNA sequencing. The operational taxonomic units (OTUs) curve shifted closer to the control group after trigonelline treatment (Figure S8A, Supporting Information), and the Venn diagram showed different OTUs between the HFpEF and Htrig (HFpEF mice received 100 mg kg^−1^ trigonelline treatment) group (Figure S87B,C), implicating that trigonelline might affect gut microbiota structure in HFpEF mice. As shown in **Figure** **6**A–C, the observed species, Chao1 (reflecting the species richness), and Simpson indices (estimating the species diversity) analysis revealed that HFpEF mice had decreased α‐diversity. Trigonelline supplementation reversed the α‐diversity in HFpEF mice. The PCoA analysis indicated that the overall gut microbial communities of the HFpEF mice were strongly altered by trigonelline (Figure 6D). To determine if altered gut microbiota participated in AMPK activation in trigonelline‐treated mice, HFpEF mice were treated with antibiotics (Abx) before oral administration of trigonelline to block gut microbiota (Figure 6E). As shown in Figure 6F–G, antibiotic treatment itself had no significant impact on AMPK signaling in the heart or liver of HFpEF mice. But antibiotic treatments markedly blocked cardiac and haptic AMPK activation in trigonelline‐treated HFpEF mice.

To further test whether circulating factors mediate the microbiota‐dependent effects of trigonelline on AMPK, we performed in vitro experiments using serum from different treatment groups. The results showed that serum from HFpEF + trigonelline mice markedly increased phosphorylation of AMPK in both cardiomyocytes and hepatocytes compared with serum from HFpEF mice, whereas this effect was abolished when serum from antibiotic‐treated HFpEF + trigonelline mice was used (Figure 6I–K).

Taken together, these data demonstrated that gut microbiota were required for trigonelline‐induced AMPK activation in HFpEF mice, and trigonelline might activate AMPK in target tissues through microbiota‐dependent circulating factors.

### 2.7 Altered Gut Microbiota were Required for the Protective Effects of Trigonelline on Metabolism and Cardiac Function of HFpEF Mice

**Figure 7:**
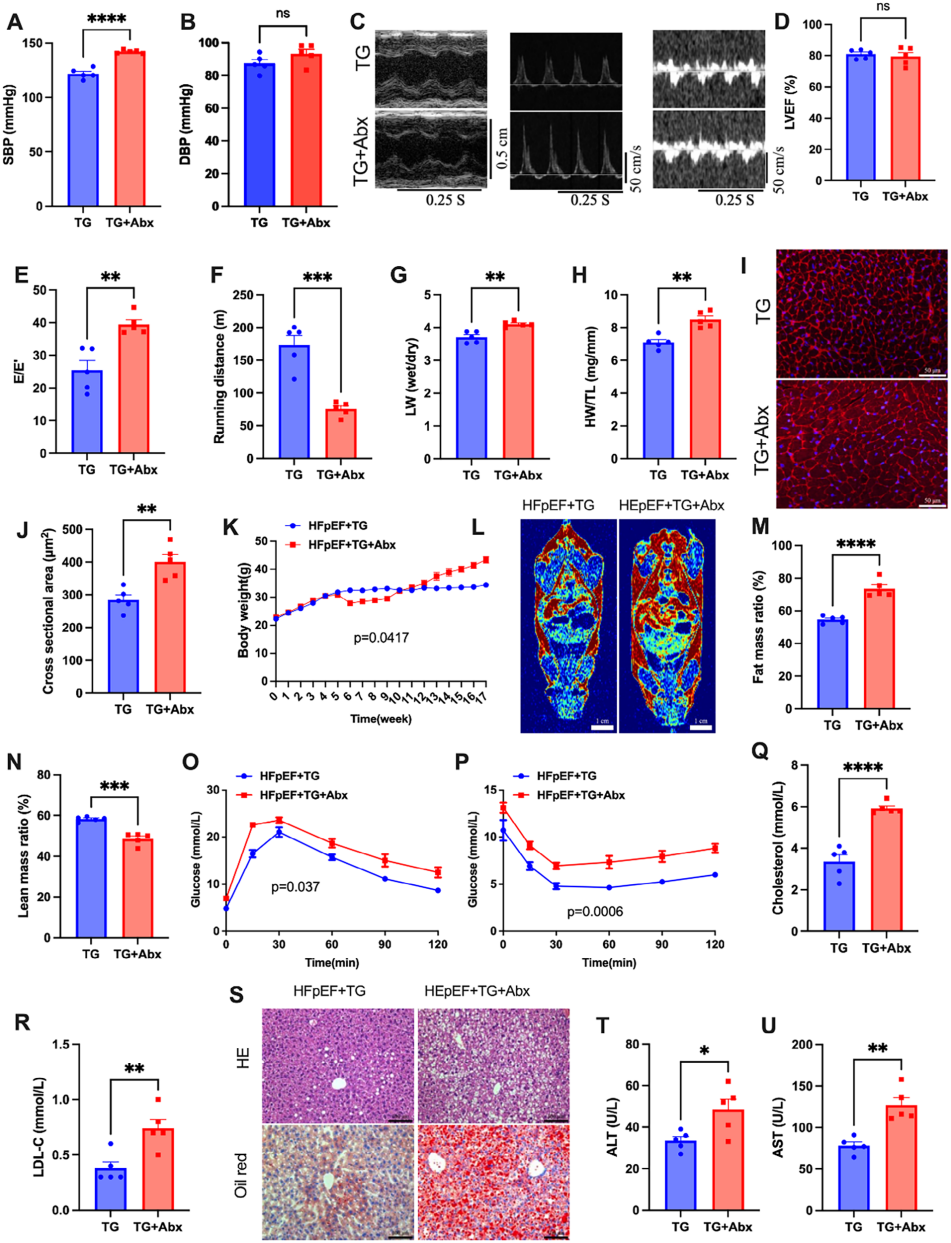
Antibiotic treatment eliminates the trigonelline‐mediated protective effects in HFpEF mice. A,B) SBP and DBP of different experimental groups were noninvasively measured by the tail‐cuff method (*n* = 5 mice per group). C) Representative echocardiography‐derived M‐mode tracings (left), pulsed‐wave Doppler (middle), and tissue Doppler (right) tracings of mice in the indicated group. D) Percent left ventricular ejection fraction (LVEF%). E) The ratio between mitral E wave and E’ wave (E/E’). F) Running distance during the exercise exhaustion test of mice. G) The ratio between wet and dry lung weight (LW). H) Ratio between heart weight and tibia length (HW/TL) (for LVEF%, E/E’ ratio, running distance, LW wet/LW dry ratio, and HW/TL ratio, *n* = 5 mice per group). I) Representative images of WGA in transversal sections of the left ventricle of mice of different experimental groups. Scale bar: 50 µm for WGA. J) WGA quantification of cardiomyocyte cross‐sectional area (*n* = 5 mice per group). K) Body weight was monitored weekly in each experimental group. L) Representative images of MRI of mice of different experimental groups. Red indicates higher fat content, green represents moderate fat content, and blue corresponds to low fat content. Scale bar: 1 cm for MRI. Fat mass M) and lean mass N) ratio of mice in the indicated groups (*n* = 5). O) Glucose tolerance tests in the indicated groups (*n* = 5). P) Insulin sensitivity tests in the indicated groups (*n* = 5). Q) Serum total cholesterol and Low‐density lipoprotein cholesterol (LDL‐C) R) in each group. S) Representative images of hematoxylin and eosin staining and oil red staining of liver from mice in each treated group, Scale bar = 100 µm. T) Serum ALT and AST U) in each group. (for total cholesterol, LDL‐C, Serum ALT, AST, *n* = 5 mice per group). Data are presented as mean ± SEM and analyzed using Student's *t*‐test. ns, no significant; ^*^ *p* < 0.05, ^**^ *p* < 0.01, ^***^ *p* < 0.001, and ^****^ *p* < 0.0001.

The gut microbiota‐dependent nature of AMPK activation would predict that the cardioprotective actions of trigonelline also required the involvement of gut microbiota. Indeed, blocking gut microbiota blunted the cardioprotective effects of trigonelline in HFpEF mice, including blood pressure (**Figure** **7**A,B), cardiac function (Figure 7C–E; Figure S9, Supporting Information), exercise tolerance (Figure 7F), lung edema (Figure 7G), and cardiomyocyte hypertrophy (Figure 7H–J). Similarly, trigonelline‐induced improvements in metabolic phenotypes, including obesity (Figure 7K–N), insulin resistance (Figure 7O,P), hyperlipidemia (Figure 7Q,R), and hepatic steatosis (Figure 7S–U) were all significantly reduced by antibiotic treatments. Blocking gut microbiota did not appear to change mice's food intake or renal function (Figure S10, Supporting Information). Thus, these cardiac function and metabolic studies reinforced the hypothesis that trigonelline acted through gut microbiota in HFpEF mice.

### 2.8 Trigonelline Downregulated Firmicutes/Bacteroidetes Ratios of Gut Microbiota in HFpEF Mice

**Figure 8:**
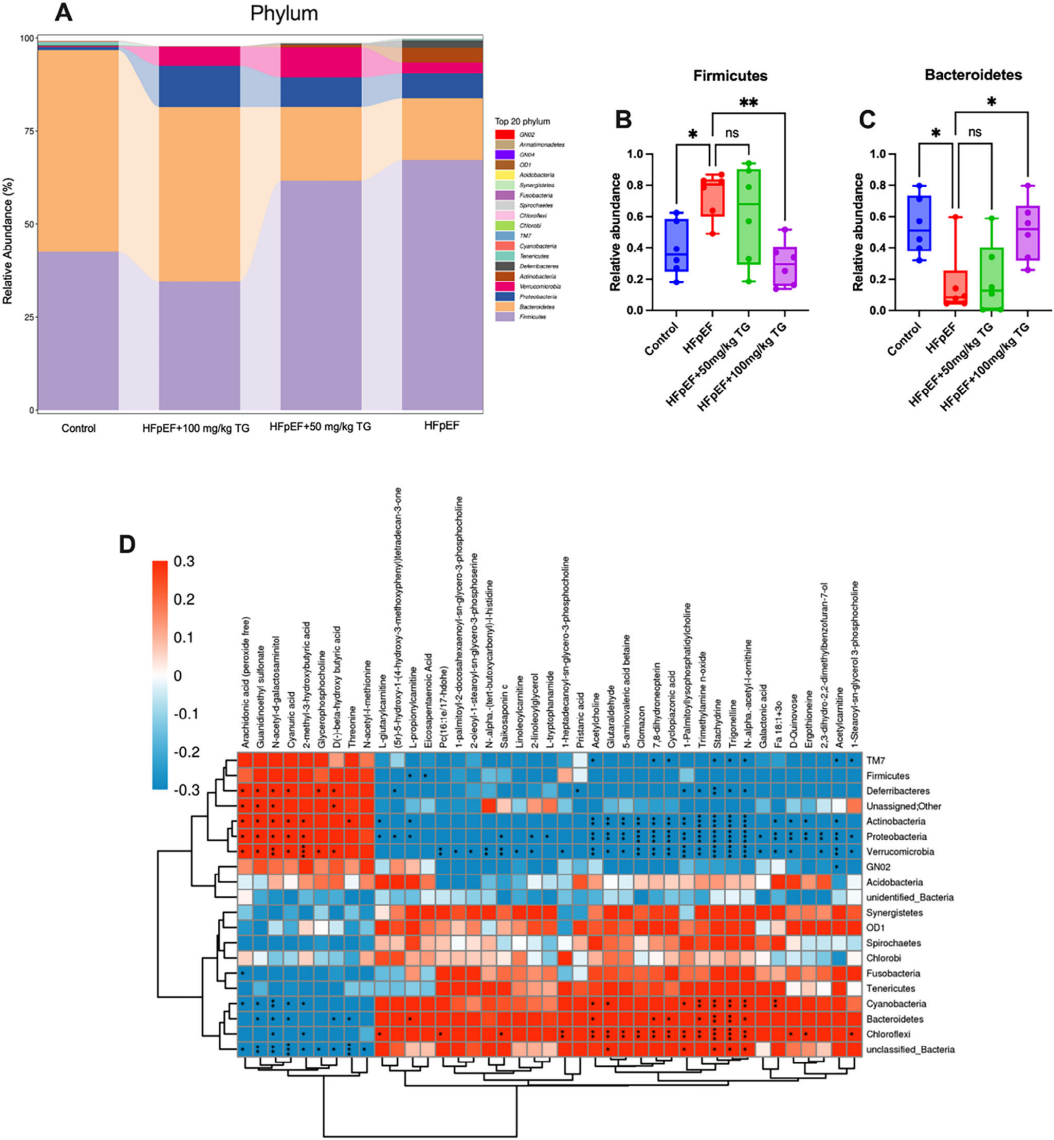
Trigonelline downregulated Firmicutes/Bacteroidetes ratios of gut microbiota in HFpEF mice. A) The relative abundance of gut bacterial phylum in each group. The relative abundance of the gut Firmicutes B) and Bacteroidetes C) in each group (*n* = 6 mice per group). D) Correlation analysis between fecal 16S rRNA sequences and metabolites by partial Spearman's correlation. For B and C, Data are presented as mean ± SEM and analyzed using one‐way ANOVA followed by Tukey's multiple comparisons test. ^*^ *p* < 0.05, ^**^ *p* < 0.01.

Finally, we strived for more details on how gut microbiota alterations might induce AMPK activation in trigonelline‐treated HFpEF mice. By using 16s rDNA sequencing, we analyzed the clustering of species composition with a particular interest in the Firmicutes/Bacteroidetes (F/B) ratios because decreased F/B ratios had been linked to AMPK activation in humans and animals.^[^ ^33^, ^34^ ^]^ As shown in Figure S8C,D (Supporting Information), the clustering of species composition and relative species abundance at the phylum and genus level showed low similarity in species composition between the control, HFpEF, and trigonelline supplementation groups. Specifically, HFpEF mice gut microbiota was differentiated by increased Firmicutes and decreased Bacteroidetes, which indicated that HFpEF mice had severe gut microbiota dysbiosis. Trigonelline reversed these by significantly downregulating Firmicutes and upregulating Bacteroidetes in HFpEF mice (**Figure** **8**A–C). Moreover, the correlation analysis (Figure 8D) showed that trigonelline was positively correlated with Bacteroidetes, again implying that trigonelline supplementation might decrease the F/B ratios in HFpEF mice. The specific information on the correlation analysis of metabolites and gut microbiota is shown in Datasets S3 and S4 (Supporting Information). These findings together suggested that altered gut microbiota with reduced F/B ratios might be involved in AMPK activation and subsequent cardioprotection in trigonelline‐treated HFpEF mice.

## 3 Discussion

In this study, we identified trigonelline as a microbiota‐dependent metabolite that ameliorates HFpEF by activating AMPK signaling. Trigonelline supplementation improved diastolic function and alleviated metabolic remodeling. These effects were abolished by antibiotic‐mediated microbiota depletion, highlighting the essential role of gut microbiota in mediating its cardioprotective actions (Graphical abstract).

Trigonelline is a plant alkaloid found in coffee and fenugreek.^[^ ^24^ ^]^ Plasma trigonelline, a coffee intake marker,^[^ ^35^ ^]^ exhibits antihyperglycemic, antihyperlipidemic, and anti‐neurodegenerative, mitochondrial protective effects.^[^ ^36^, ^37^, ^38^, ^39^, ^40^ ^]^ As some serum metabolome associates with cardiac function and myocardial remodeling in human^[^ ^41^ ^]^ and our metabolomic analyses revealed that trigonelline is the most decreased metabolite in the heart of HFpEF mice. Therefore, we systematically evaluated the effects and potential mechanisms of oral supplying trigonelline in HFpEF mice. We found that trigonelline supplementation alleviated the manifestations of HFpEF in mice, including exercise intolerance, lung edema, cardiac dysfunction, hypertrophy, and metabolic disorders. In fact, some early clinical studies already reported that trigonelline levels were found to be decreased in obese and type 2 diabetic patients.^[^ ^42^, ^43^ ^]^ Together, these findings suggest that HFpEF might be treated with trigonelline.

Loss of AMPK activity is a hallmark of cardiometabolic syndromes.^[^ ^12^, ^44^ ^]^ Previous studies have demonstrated that impaired AMPK activity is involved in the development of HFpEF in mice.^[^ ^45^, ^46^ ^]^ Moreover, activating the AMPK pathway with metformin was reported to attenuate HFpEF‐associated cardiac injury.^[^ ^45^ ^]^ The potential mechanisms responsible for the protective role of AMPK activation including enhancing fatty acid oxidation, stimulating mitochondrial biogenesis, regulating autophagic flux and suppresses inflammatory and fibrotic signaling.^[^ ^12^ ^]^ And, various evidence suggests GSK‐3β and AMPK exert opposing roles in cellular energy homeostasis and involved into many metabolic abnormal phenotypes.^[^ ^47^ ^]^ Therefore, we speculated that trigonelline might function via regulating the AMPK‐GSK‐3β axis. We observed that trigonelline supplementation induced phosphorylation of AMPK and GSK‐3β in the heart and liver of HFpEF mice. The pretreatment of mice with an AMPK inhibitor abolished the protective effects of trigonelline in HFpEF mice. Since GSK‐3β and AMPK exert antagonistic effects on cellular energy homeostasis, this finding suggests that trigonelline may engage an AMPK‐GSK‐3β signaling axis to confer additional metabolic and functional benefits. Taken together, these data establish a more comprehensive mechanistic framework in which trigonelline‐mediated AMPK activation orchestrates multiple downstream pathways and GSK3β regulation to ameliorate HFpEF. Even so, one may reckon whether an AMPK agonist may do similar benefits to HFpEF hearts. However, the pan‐AMPK activator has been shown to induce cardiac hypertrophy.^[^ ^48^ ^]^ Different from pan‐AMPK activator, trigonelline induced AMPK activation in hearts and livers but those in skeleton muscle or kidneys. Such a feature may render trigonelline advantages in treating cardiovascular metabolic diseases including HFpEF.

Unexpectedly, trigonelline did not directly activate AMPK in cultured cardiomyocytes or haptic cells, suggesting that trigonelline influences AMPK signaling indirectly in vivo. Gut microbiota is vital in developing metabolic syndrome via regulating energy balance.^[^ ^23^, ^49^ ^]^ Many studies have linked gut microbiota dysbiosis to cardiovascular diseases.^[^ ^50^ ^]^ Gut microbiota dysbiosis also exists in HFpEF patients, and a recent study reported that gut microbiota may regulate the AMPK pathway in obese mice.^[^ ^51^, ^52^ ^]^ In the present study, we found that trigonelline modified the gut microbiota in HFpEF mice, as evidenced by the increased α‐diversity and decreased Firmicutes and Bacteroidetes ratios, demonstrating a significant association between trigonelline supplementation and a reshaped gut microbiota composition. A pivotal question is whether these microbial changes are a direct driver or a secondary consequence of improved host metabolism. Our functional experiments provide critical insight into this mechanism. The observation that the protective effects of trigonelline on AMPK activation, systemic metabolic disturbances, and cardiac function were substantially attenuated following the ablation of gut microbiota by broad‐spectrum antibiotics strongly suggests that an intact gut microbiota is indispensable for trigonelline's efficacy. This finding positions the gut microbiota not merely as a passive associate but as a necessary mediator in the therapeutic pathway of trigonelline. One possible explanation for that is trigonelline, as a dietary‐derived alkaloid, directly interacts with microbial taxa in the gut lumen, thereby selectively modulating their metabolic activity or growth. However, further research is required to confirm this conclusion.

Our study reveals that trigonelline can regulate AMPK by modifying the gut dysbiosis microbiome in HFpEF mice. Nevertheless, several limitations should be acknowledged. First, we did not perform a comprehensive characterization of the gut microbiome and its associated metabolites. Future studies using metagenomic sequencing to delineate specific microbial taxa, and targeted analyses of short‐chain fatty acids, bile acids, or NAD⁺ precursors that could mechanistically link microbial metabolism to AMPK activation. Second, gut microbiota depletion in our study was achieved by a two‐week broad‐spectrum antibiotic regimen. Although this protocol is widely employed and known to induce long‐lasting dysbiosis, we cannot exclude the possibility that partial recolonization occurred during the subsequent treatment period, which may influence the interpretation of our findings. Third, we did not perform fecal microbiota transplantation or germ‐free mouse experiments, which would provide a more definitive model to dissect the microbiota‐dependent mechanisms of trigonelline. Gender might also be an influencing factor to be considered.^[^ ^53^ ^]^ Future work using these models, in combination with longitudinal microbiome profiling, will be needed to confirm the sustained role of gut microbiota in mediating the cardioprotective effects of trigonelline.

## 4 Conclusion

In conclusion, trigonelline improves metabolic disorders and cardiac function via activating the AMPK pathway in a gut microbiome‐dependent manner, suggesting that trigonelline can be a novel therapeutic agent for HFpEF.

## 5 Experimental Section

A full detailed section of the Experimental Section can be found in the Supporting Information online.

### Ethics Statement

The C57BL/6J mice were purchased from the Chengdu Gempharmatech company. Only male (6‐week‐old) mice were used for the experiments. All animal experiments were approved by the Institutional Animal Care and Use Committee of the Chongqing Medical University, China (IACUC‐CQMU‐2023‐12097). All procedures conformed to the guidelines from the NIH Guide for the Care and Use of Laboratory Animals.

### Animal Experiments

Mice were maintained in specific pathogen‐free facilities and a vivarium with unrestricted access to food and water with a 12‐h light/dark cycle at 22 °C. L‐NAME (0.5 g L^−1^, Sigma–Aldrich) was added to the drinking water for the specified durations after adjusting the pH to 7.4. After five weeks of high‐fat diet (HFD, 60% kcal from fat) and L‐NAME (0.5 g L^−1^ in drinking water) treatment, the mice were exposed to 50 or 100 mg kg^−1^ day^−1^ trigonelline chloride (MedChemExpress, HY‐N0415) dissolved in drinking water for eight weeks. To test the role of AMPK in the therapeutic effects of trigonelline in vivo, HFpEF mice were pre‐treated with AMPK inhibitor Compound C (CC) (MedChemExpress, HY‐13418, 5 mg kg^−1^ day^−1^) or vehicle for 1 week.^[^ ^28^ ^]^ The mice were then treated with vehicle, trigonelline (100 mg kg^−1^ day^−1^), or trigonelline (100 mg kg^−1^ day^−1^) in combination with CC (5 mg kg^−1^ day^−1^) for another 11 weeks. At the end of the experiments, the body composition was measured using Newman MRI (Newman MRI Systems, China).

In the microbiota depletion studies, gut microbiota depletion was achieved by administering mice a broad‐spectrum antibiotic cocktail (1 g L^−1^ ampicillin, 1 g L^−1^ metronidazole, 1 g L^−1^ neomycin, and 0.5 g L^−1^ vancomycin) in autoclaved (tap) drinking water for two weeks.^[^ ^54^ ^]^ The drinking water was replaced twice weekly. After depleting the gut microbiota, mice were treated with trigonelline (100 mg kg^−1^ day^−1^) for another 11 weeks. For echocardiographic assessments, anesthesia was induced with 3% isoflurane and maintained at 1.0%–1.5% by inhalation. For tissue collection, mice were anesthetized with 5% isoflurane and then euthanized using high‐concentration carbon dioxide by inhalation.

### Cecal DNA Extraction and 16S rRNA Gene Analyses

Fresh stool samples were collected from animals in an empty cage without bedding for 15 min and stored at −80 °C until analysis. The OMEGA Soil DNA Kit (M5635‐02) (Omega Bio‐Tek, Norcross, GA, USA) was used to extract the cecal DNA. The V3–V4 region of the bacterial 16S rRNA was amplified using the primers 338F (F:5′‐ACTCCTACGGGAGGCAGCA‐3′ and 806R R:5′‐GGACTACHVGGGTWTCTAAT‐3′). Sequencing was performed using the Illumina MiSeq platform with the MiSeq Reagent Kit v3. Microbiome bioinformatics was performed with QIIME 2 2019.4,^[^ ^55^ ^]^ while the OTU clustering procedure followed the Vsearch (v2.13.4) pipeline^[^ ^25^ ^]^ described here (https://github.com/torognes/vsearch/wiki/VSEARCH‐pipeline).

### Metabolomics Analysis Based on LC/MS

The heart tissues were frozen in liquid nitrogen immediately after dissection. Then, the tissues were cut on dry ice (≈80 mg). LC‐MS/MS analysis was performed using UHPLC (1290 Infinity LC, Agilent Technologies) coupled to a quadrupole time‐of‐flight (AB Sciex TripleTOF 6600) at Shanghai Applied Protein Technology Co., Ltd. The raw MS data (wiff.scan files) were converted to MzXML files using ProteoWizard MSConvert before being imported into freely available XCMS software. For peak picking, the following parameters were used: centWave m/z = 10 ppm, peakwidth = c (10, 60), and prefilter = c (10, 100). For peak grouping, bw = 5, mzwid = 0.025, and minfrac = 0.5 were used. After sum‐normalization, the processed data were analyzed by R package (ropls). VIP > 1 and *p*‐value < 0.05 were used to screen significantly changed metabolites.

### Statistical Analysis

All data are expressed as mean ± SEM. The Student's *t*‐test was employed to analyze differences between the two groups. One‐way or two‐way ANOVA was applied for multiple comparisons. Statistical differences with *p*‐values less than 0.05 were considered significant.

## Conflict of Interest

The authors declare no conflict of interest.

## Supporting information



Supporting Information

## Data Availability

The data that support the findings of this study are available in the supplementary material of this article.
