# Supplementary material for: Trigonelline Improves Metabolism and Cardiac Function of HFpEF Mice Via Gut Microbiome Alterations‐Mediated AMPK Activation
Source: Adv Sci (Weinh). 2025 Nov 5;13(5):e13956. doi: 10.1002/advs.202513956 (PMC12849935; doi:10.1002/advs.202513956)
Supplement: Supplementary file 1 — Supporting Information [file ADVS-13-e13956-s001.docx]

**Supplementary methods**

**Echocardiography and Doppler imaging**

Transthoracic echocardiography was performed using a VINNO 6 VET ultrasound system in small animals. Anesthesia was induced by 3% isoflurane, which was confirmed by a lack of response to firm pressure applied to one of the hind paws. During echocardiogram acquisition under body temperature-controlled conditions, isoflurane was reduced to 1.0%–1.5% and adjusted to maintain heart rate in the range of 400–500/min. Finally, all mice were recovered from anesthesia. All parameters were measured at least three times, and the averages were calculated.

**Tail cuff blood pressure recordings**

Blood pressure was measured noninvasively in conscious mice using the tail-cuff method. The animals were placed in holders on a 37 °C platform and recorded under steady-state conditions after acclimatization. Blood pressure was recorded for at least four consecutive days, and readings were averaged from at least eight measurements per session.

**Exercise exhaustion test**

After three days of treadmill acclimatization, an exhaustion test was performed. The animals ran treadmill inclination (20°) on a treadmill (Columbus Instruments), starting at a warm-up speed of 5 m/min for 4 min, after which the speed was increased to 14 m/min for 2 min. Every subsequent 2 min, the speed was increased by 2 m/min until the animals were exhausted. Exhaustion was defined as the inability of the animal to return to running within 10 s of direct contact with an electric stimulus grid.

**Glucose tolerance and insulin sensitivity test**

After fasting for 8 h, mice received a 2 g/kg D-glucose bolus intraperitoneally. Blood glucose levels were measured with a glucometer (Roche) at 0, 15, 30, 60, 90, and 120 min after injection. For the insulin sensitivity test, mice were fasted for 4 h, and blood glucose levels were measured at 0, 15, 30, 60, 90, and 120 min after 0.75 IU/kg insulin injection.

**Histopathologic analyses**

For histopathological analyses, lipid droplets were visualized by Oil Red O (Sigma-Aldrich) staining of Tissue-Tek O.C.T. Compound (Servicebio)-embedded frozen liver tissues. Masson’s trichrome staining was performed following the manufacturer’s protocol using a Solarbio kit (cat No. G1340). The cardiomyocyte cross-sectional area was measured by wheat germ agglutinin (WGA) staining (Alexa Fluor™ 594 conjugated; Invitrogen #W11262) at 5 μg/mL in the dark for 30 min. Histopathological images were captured using a Leica IB4 microscope. Images were quantified using Image-Pro Plus 6.0.

**Western Blots**

Briefly, proteins from mouse heart or liver tissues were extracted and quantified using a BCA Protein Assay Kit. The protein samples were separated using 8%–10% SDS-PAGE gels and then transferred to polyvinylidene difluoride membranes. After blocking with 5% skim milk, membranes were incubated with the indicated primary antibodies overnight at 4 ℃ and then with secondary HRP-conjugated antibodies (Invitrogen, Goat anti-rabbit #31460, 1:10000; Goat anti-mouse #31430, 1:10000) for 90 min at room temperature. The protein expression signals were detected on a Bio-Rad Imaging System. The following primary antibodies were used: p-AMPK (CST, 2535S; 1:1000), AMPK (CST, 5831S; 1:1000), p-GSK-3β (CST, 5558T; 1:1000), GSK-3β (CST, 12456T; 1:1000), and GAPDH (Proteintech, 60004-1-Ig; 1:20000).

**
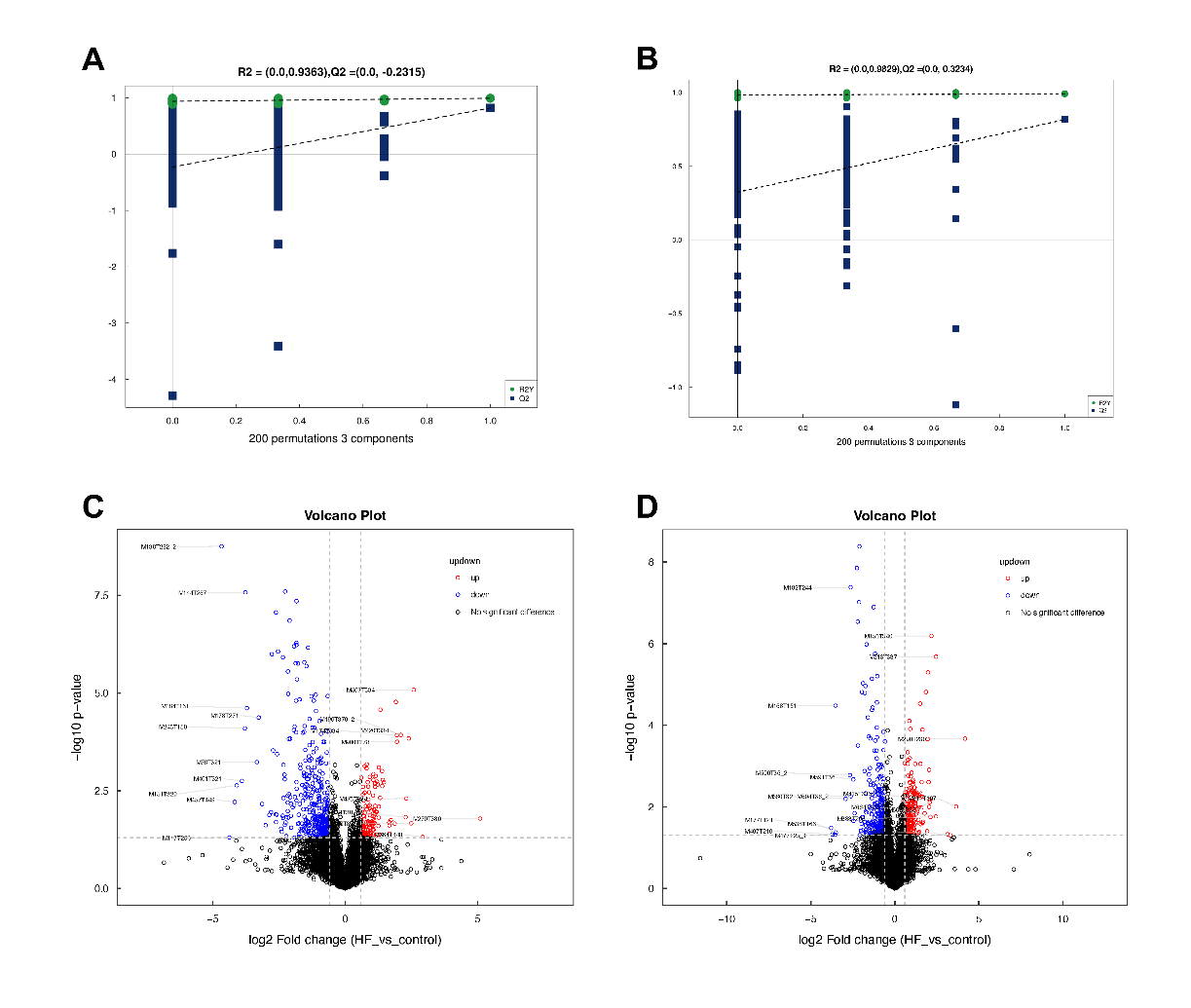
**

**Figure S1. Metabolomics profiling identifies various varying metabolites in the heart tissues of HFpEF mice.** (A–B) The validity of PLS-DA was confirmed by permutation tests in positive and negative modes. (C–D) The volcano plots of the identified differential metabolites between the control and HFpEF group in positive and negative modes.


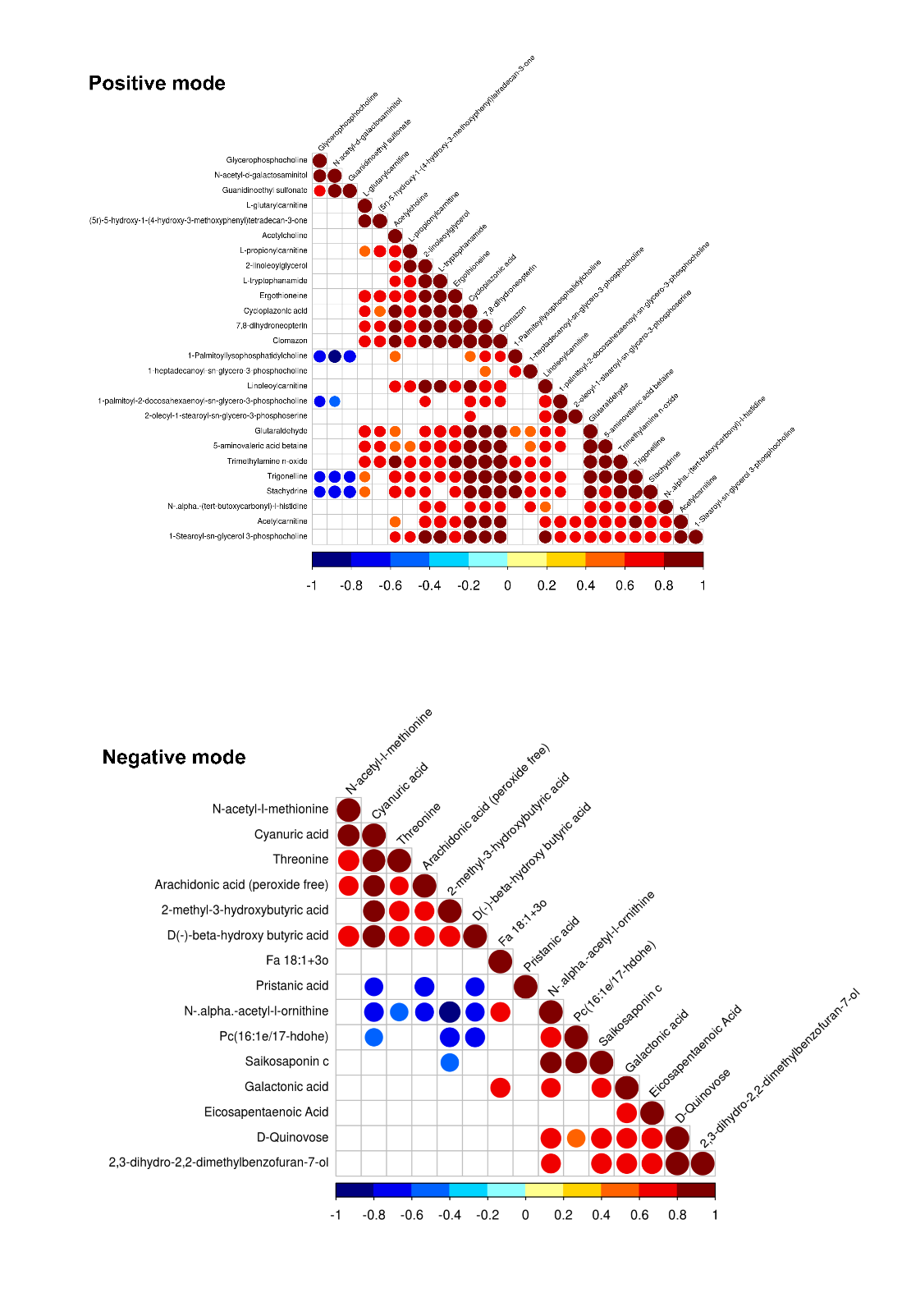


**Figure S2. Correlation analysis of the identified differential metabolites by partial Spearman’s correlation.** Correlation analysis of the identified differential metabolites in (A) positive and (B) negative modes (n = 6).


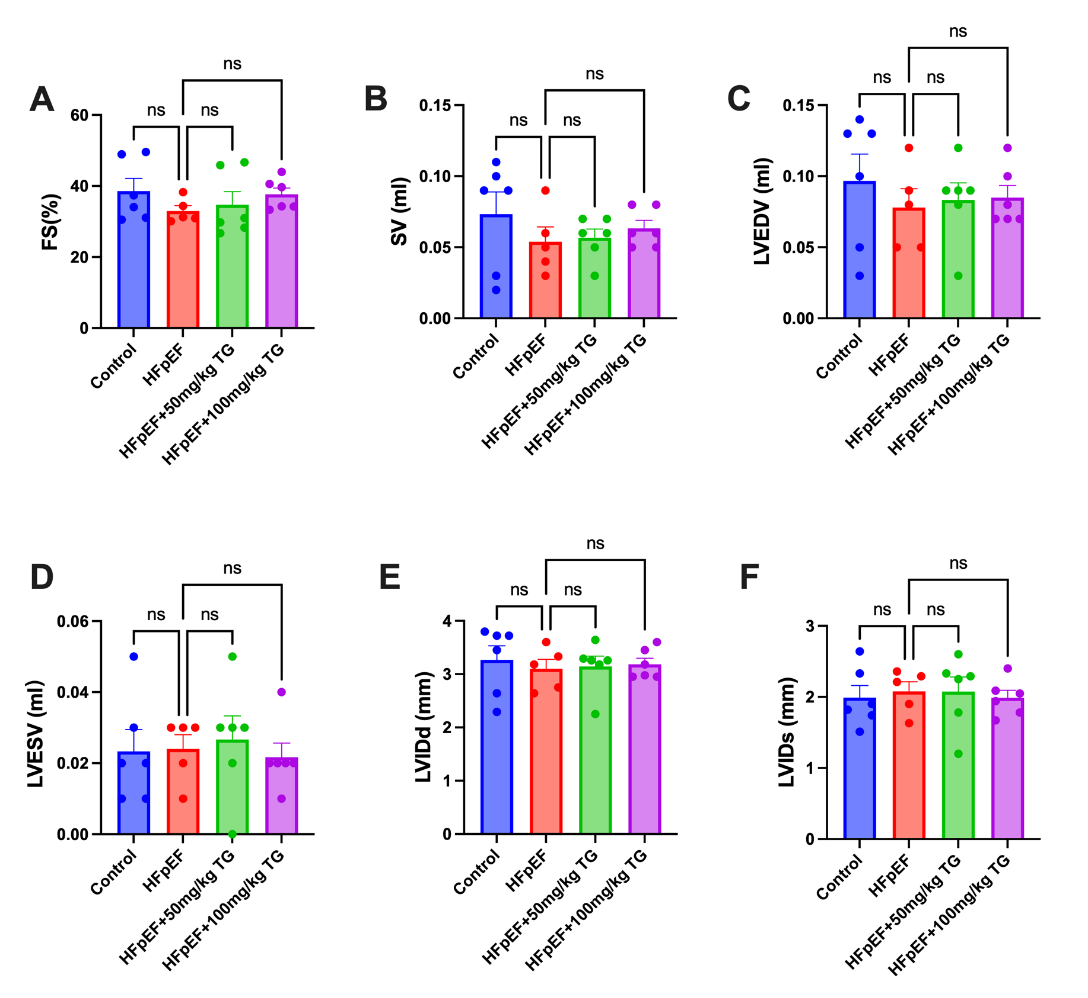


**Figure S3. The effects of trigonelline supplementation on cardiac dysfunction in HFpEF mice.** Left ventricular fractional shortening (FS%) (A), stroke volume (B), left ventricular end-diastolic volume (LVEDV, C), left ventricular end-systolic volume (LVESV, D), left ventricular internal diameter at end-diastole (LVIDd, E), and left ventricular internal diameter at end-systole (LVIDs, F) were assessed in each group (n = 6 mice per group). Data are presented as mean ± SEM and analyzed using one-way ANOVA followed by Tukey’s multiple comparisons test. ns, not significant


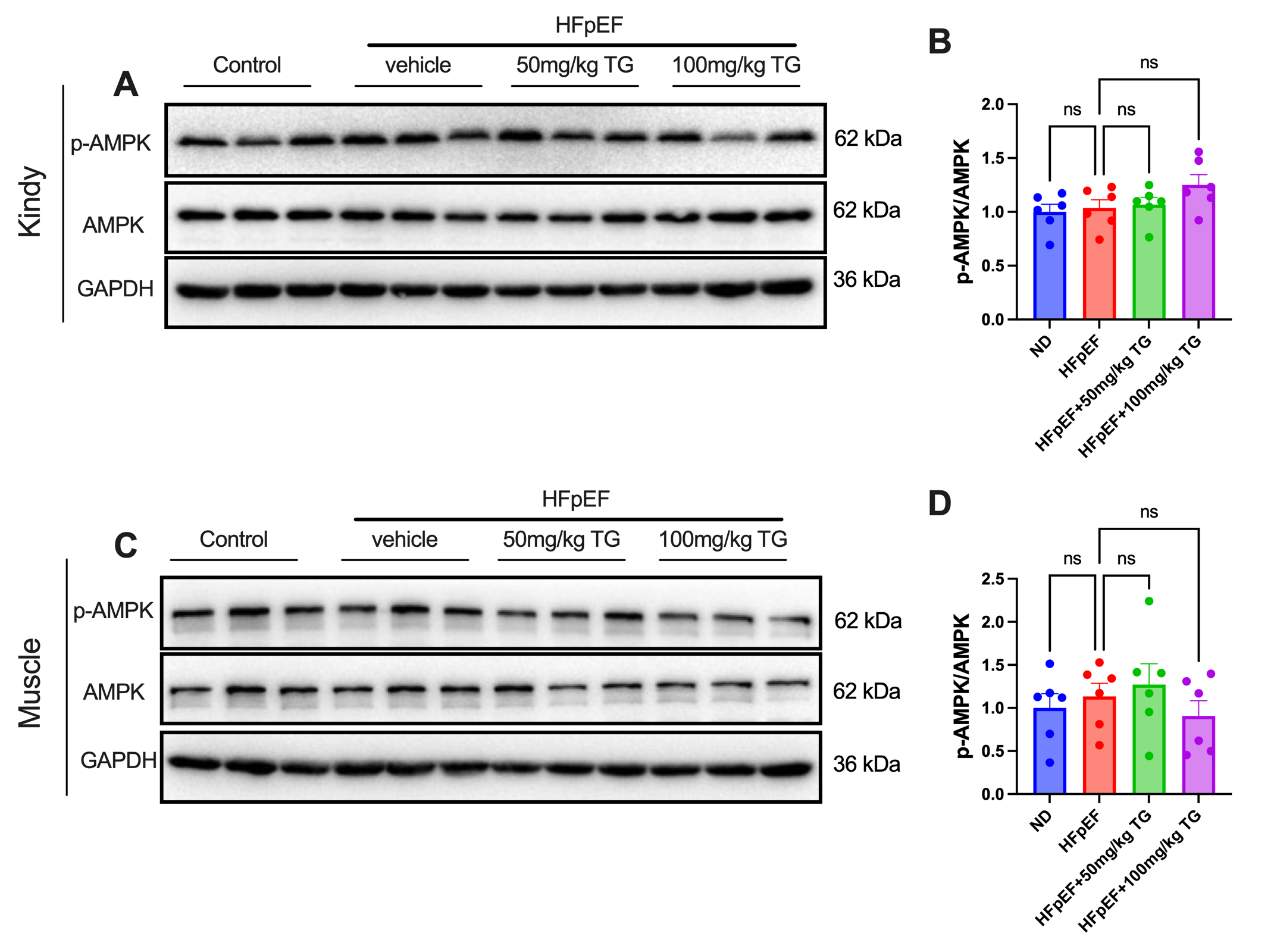


**Figure S4. Trigonelline does not actives the AMPK pathway in the kidney and muscle tissues of HFpEF mice.** (A) Representative immunoblot images of total and phosphorylated AMPK of kidney tissues from HFpEF mice. (B) Quantification of p-AMPK/AMPK ratio (n = 6). (C) Representative immunoblot images of total and phosphorylated AMPK in muscle tissues from HFpEF mice. (D) Quantification of p-AMPK/AMPK ratio (n = 6). Data are presented as mean ± SEM and analyzed using one-way ANOVA followed by Tukey’s multiple comparisons test. ns, no significant.


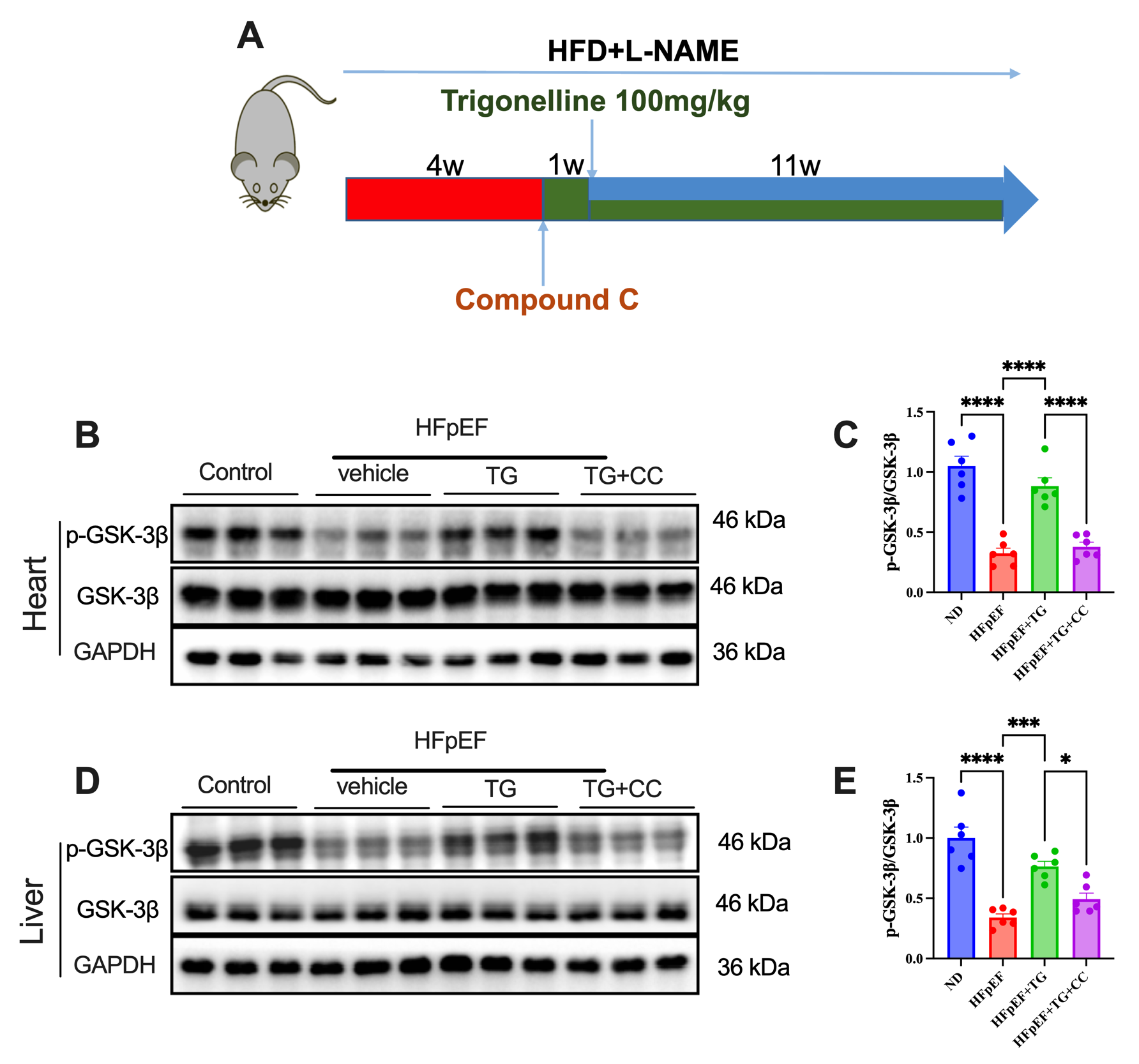


**Figure S5. AMPK activation was required for the cardioprotective effects of trigonelline in HFpEF mice.** (A) Scheme for the experimental strategy in HFpEF mice treated with trigonelline and Compound C. (B-E) Representative immunoblot images of total and phosphorylated GSK-3β in the heart and liver tissues from HFpEF mice receiving trigonelline with or without AMPK inhibitor. Quantificated results were shown in C and E (n = 6 mice per group). Data are presented as mean ± SEM and analyzed using one-way ANOVA followed by Tukey’s multiple comparisons test. ns, no significant; **P < 0.01, ****P < 0.0001.

**
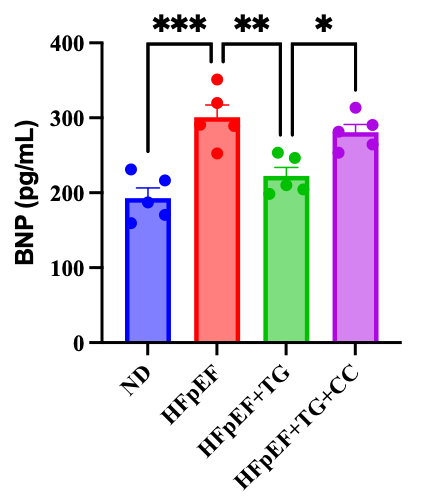
**

**Figure S6.** The BNP in serum of mice in different groups. (n = 5 mice per group). Data are presented as mean ± SEM and analyzed using one-way ANOVA followed by Tukey’s multiple comparisons test.

**
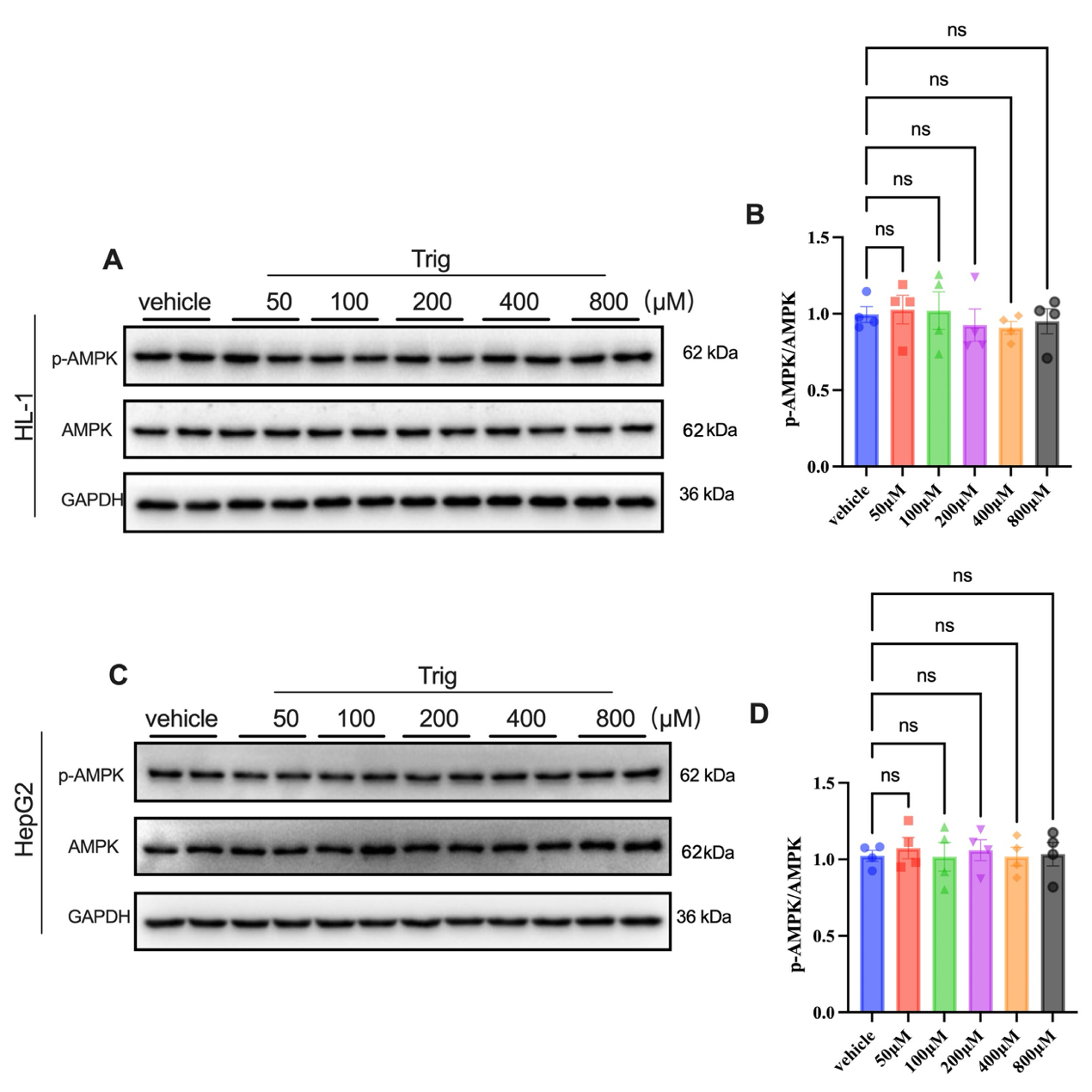
**

**Figure S7. Trigonelline does not directly actives the AMPK pathway *in vitro*.** (A) Representative immunoblot images of total and phosphorylated AMPK of HL-1 cell lines under various trigonelline treatments. (B) Quantification of cardiac p-AMPK/AMPK ratio (n = 4). (C) Representative immunoblot images of total and phosphorylated AMPK in HepG2 cell lines under various trigonelline treatments. (D) Quantification of cardiac pAMPK/AMPK ratio (n = 4). Data are presented as mean ± SEM and analyzed using one-way ANOVA followed by Tukey’s multiple comparisons test. ns, no significant.

**Figure S8. 16s rDNA sequencing profiling in HFpEF mice treated with trigonelline.**

(A) Rank abundance curve. The abscissa represents the ranking level of the operational taxonomic units (OTUs) number, and the ordinate represents the relative percentage content of the OTU number. The abscissa position of the extension end point of the sample curve is the number of OUT. (B) Venn diagram illustrating the four groups of differentiated OTUs. (C) The relative ASV/OUT abundance of gut bacterial phylum in each region in Venn diagram. (D) The heatmap of the gut bacterial genus in each group. Data are shown as the means with minimum to maximum. HF, HFpEF; Ltrig, HFpEF+50 mg/kg trigonelline treatment. Htrig, HFpEF+100 mg/kg trigonelline treatment.

**
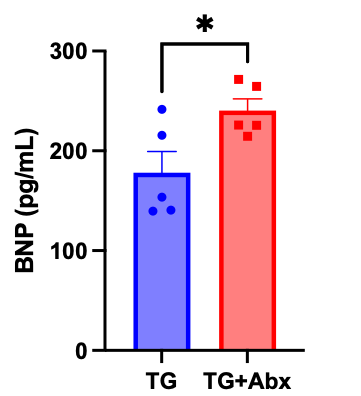
**

**Figure S9. Blocking gut microbiota blunted the cardioprotective effects of trigonelline in HFpEF mice.** The BNP in serum of mice in different groups. (n = 5 mice per group). Data are shown as mean ± SEM and analyzed using Student’s t-test.

**
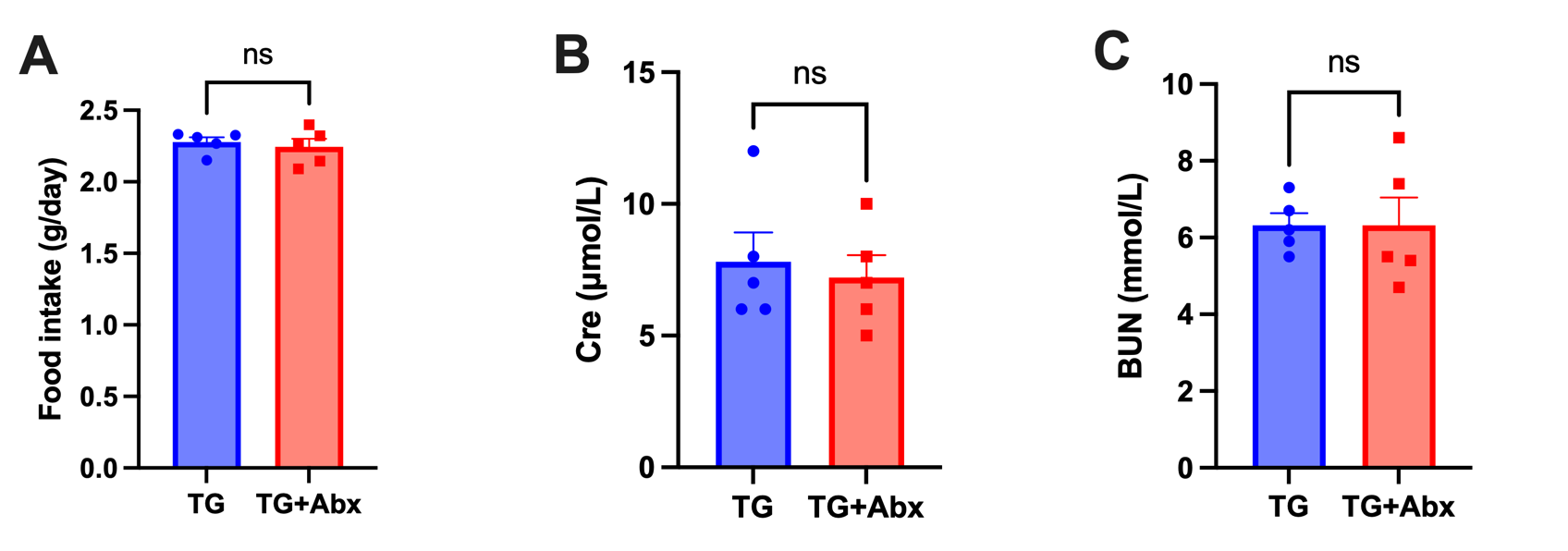
**

**Figure S10. Effects of addition of antibiotics on food intake and renal function.** (A) Food intake of mice of different experimental groups per day. (B) Serum Cre in each group. (C) Serum BUN in each group (n = 5 mice per group). Data are presented as mean ± SEM and analyzed using Student’s t-test. ns, no significant.
